# Cotton genetic mapping for plant biotechnology: from markers to graph pan-genomes and sustainable breeding

**DOI:** 10.3389/fpls.2026.1825852

**Published:** 2026-05-12

**Authors:** Ibrokhim Y. Abdurakhmonov

**Affiliations:** Center of Genomics and Bioinformatics, Academy of Sciences of Uzbekistan, Tashkent, Uzbekistan

**Keywords:** cotton, genetic mapping, GWAS, marker-assisted selection, pan-genome, plant biotechnology, polyploidy

## Abstract

Cotton improvement remains a major challenge in plant biotechnology because stable yield, fiber quality, and stress resilience must be delivered from complex allopolyploid genomes under variable environmental conditions. Genetic mapping has progressively transformed this challenge into deployable breeding knowledge, advancing from sparse marker systems to high-density SNP arrays, reference genomes, and, more recently, structural-variation-aware pan-genomes and graph genomes. This review argues that cotton’s genomic complexity, homoeolog redundancy, and strong genotype-by-environment interactions have driven methodological innovation rather than the simple transfer of approaches developed in other crops. It synthesizes advances in linkage mapping, GWAS, eQTL analysis, fine mapping, functional validation, and marker deployment, with emphasis on how these tools have enabled breeder-ready assays, marker-assisted selection, and emerging predictive breeding frameworks. It further examines how cotton mapping contributes to sustainable intensification by enhancing disease resistance, abiotic resilience, fiber value, and trait-stacking efficiency. Finally, this review highlights unresolved challenges in homoeolog-aware inference, structural variant genotyping, phenotyping throughput, and environment-aware prediction. It outlines a next-phase agenda in which graph genomes, scalable validation, and climate-informed models become central to cotton biotechnology.

## 1 Introduction

Cotton is a globally important fiber and oilseed crop whose improvement remains unusually challenging because economically important traits must be optimized within complex allotetraploid genomes under variable environments. Its two dominant cultivated species, *Gossypium hirsutum* and *G. barbadense*, are allotetraploids (AADD, 2n = 4x = 52) that formed approximately 1–2 million years ago through hybridization between A- and D-genome diploids (Jiang et al., 1998). The crop’s defining commercial trait, spinnable fiber, arises from single-celled epidermal trichomes that follow a developmental program that is both robust and highly sensitive to environmental conditions. Breeders have long exploited visible variation in lint yield, fiber length, strength, and micronaire, but converting these phenotypes into reliable genetic decisions has remained difficult.

Polyploid genomes complicate mapping by introducing homoeologous loci that can confound marker assignment while still contributing distinct trait effects. Early work by Jiang et al. (1998) showed that polyploid formation created new opportunities for response to selection, not simply through gene duplication, but through regulatory redundancy that selection could subsequently repartition. At the same time, fiber traits are often determined within narrow developmental windows, whereas field performance reflects season-long interactions among genotype, stress, and management. This combination made cotton a stringent test case for genetic mapping. In response, the field progressed through successive improvements in tools and study design to increase resolution, robustness, and reproducibility.

This review focuses on cotton-specific constraints, particularly polyploid-aware inference, field-relevant reproducibility across environments, and the validation-to-deployment pipeline that converts loci into breeder-ready assays and cultivars. In doing so, it highlights how cotton differs from broader plant-wide mapping trajectories in its constraints, resources, and routes to breeding application.

**Figure 1:**
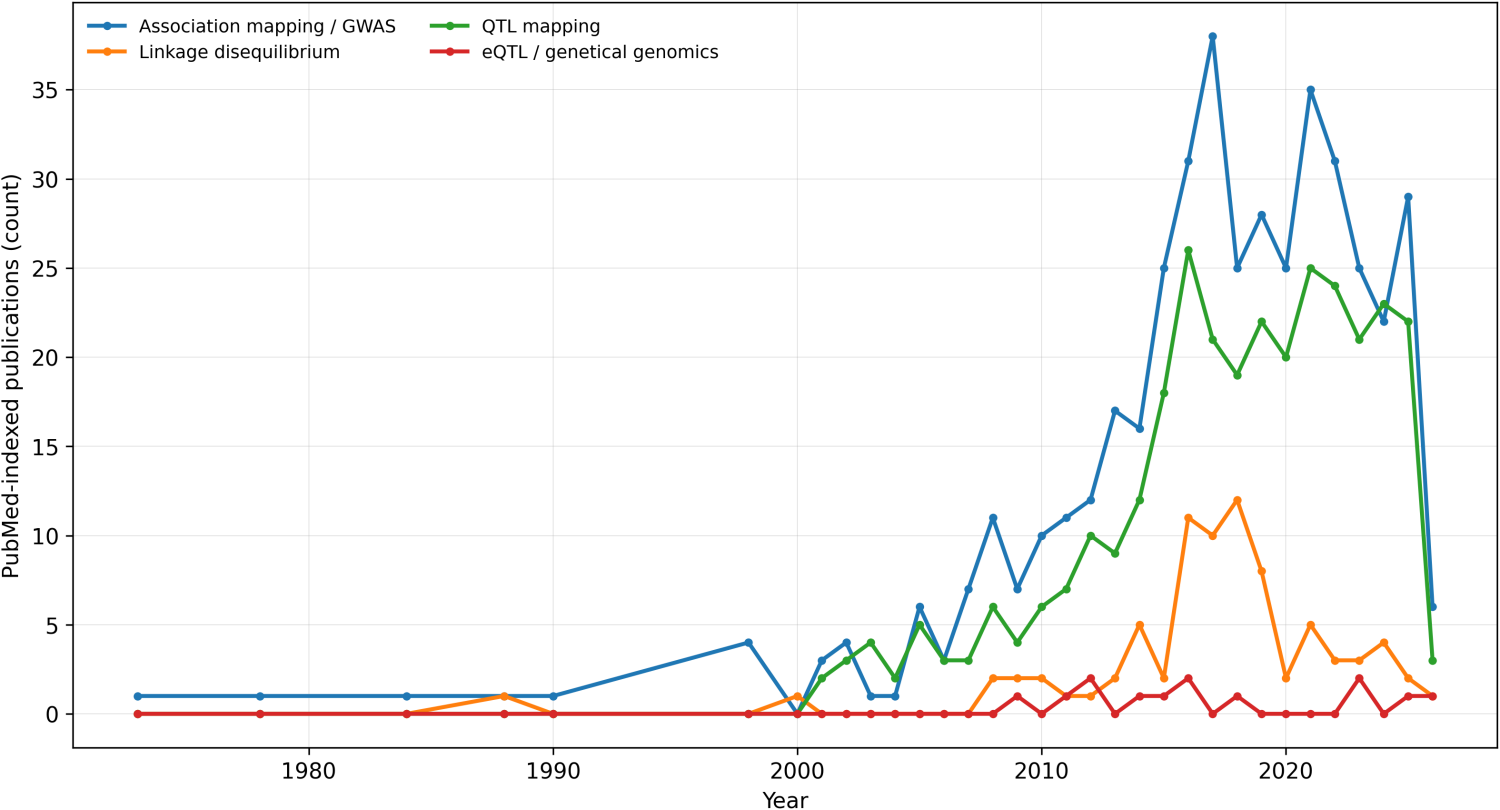
Cotton genetic mapping literature over time. PubMed-indexed publication counts per year for cotton QTL mapping, association mapping/GWAS, eQTL/genetical genomics, and linkage disequilibrium studies. The curves visualize phase changes: the QTL era, the rise of population-structure-aware association, and the emergence of genomics-enabled regulatory and pan-genome frameworks.

Two organizing frameworks anchor this review. First, publication chronologies show that cotton mapping has shifted from linkage and QTL studies to population-scale association and regulatory mapping, and more recently to structural-variation and pan-genome frameworks (**Figure 1**). Second, progress in cotton mapping has consistently depended on aligning technological advances with breeder-relevant trait priorities and deployment pathways. The major milestones in this five-decade trajectory are summarized in **Table 1**, which provides a roadmap for the sections that follow.

**Table 1:** Milestones in cotton genetic mapping, framed as challenges and solutions.

Era	Period	Core challenge	Mapping innovation	Cotton-specific outcome	Key references
Foundations	1970s–1990s	Quantitative traits + polyploid complexity obscure inheritance	Early quantitative/cytogenetic framing; recognition of polyploid selection dynamics	Trait dissection problems defined; rationale for marker-based mapping	Lee, 1973; Jiang et al., 1998
Early linkage mapping	~2000–2006	Limited polymorphism in elite pools; sparse maps	RFLP/SSR linkage maps; first-generation QTL scans	Initial QTL regions for fiber strength/yield; first MAS targets	Zhang et al., 2002, Zhang et al., 2003; Park et al., 2005
Multi-environment QTL era	~2006–2012	QTL instability across environments; polygenic traits	Multi-environment QTL; meta-QTL; higher density	Robust QTL clusters; clearer trait modules	Yu et al., 2013; Said et al., 2013
Population-aware association mapping	~2008–2017	Confounding by structure; LD heterogeneity	Linkage disequilibrium (LD) characterization; mixed models; diversity panels	Reproducible marker–trait associations; germplasm-aware targets	Abdurakhmonov and Abdukarimov, 2008; Abdurakhmonov et al., 2008, Abdurakhmonov et al., 2009; Abdullaev et al., 2017
Standardized SNP/GWAS era	~2015–2021	Harmonizing loci across studies	63K/80K arrays; panel GWAS	Comparable discoveries across programs	Hulse-Kemp et al., 2015; Cai et al., 2017; Sun et al., 2017
Genomics and regulatory mapping	~2012–2021	Homoeolog ambiguity; regulatory variation	Reference genome; transcriptomes; eQTL	From loci to mechanisms in fiber/stress	Zhang et al., 2015; Wang et al., 2017a; Claverie et al., 2012
SV/pan-genome era	~2023–present	Reference bias; SV/Presence-Absence Variant (PAV) as causal variants	SV-based pan-genomes; graph genomes	SV-aware trait discovery; better diversity representation	Jin et al., 2023; Zhang et al., 2026; Yang et al., 2026
Deployment era	ongoing	Multi-trait assembly + G×E	Marker pipelines; environment-smart testing; early GS frameworks	Cultivar delivery with quality + adaptation gains	Malik et al., 2014; Darmanov et al., 2022

## 2 Classical genetics before dense markers

Before the advent of dense molecular markers, cotton genetics during the 1970s–1990s was shaped by two central realities. First, many agronomic traits were clearly heritable, but heritability alone was not actionable at locus-level resolution. Second, polyploidy both expanded and obscured variation: when multiple homoeologous loci contributed to a trait, classical segregation analysis often provided only limited guidance on where selection should act.

A representative pre-marker trait was seed gossypol, whose inheritance could be analyzed quantitatively even within early genetic frameworks (Lee, 1973). However, this insight did not easily translate into breeding pipelines because the relevant loci could not yet be identified or tracked. This gap between recognizing that variation was genetic and being unable to identify the responsible chromosomal segments defined the central limitation of the era.

Cotton’s evolutionary biology offered a deeper explanation for this difficulty. Polyploid formation could create new avenues for response to selection, not simply by duplicating genes, but by generating regulatory and functional redundancy that selection could later repartition (Jiang et al., 1998). Even so, conceptual understanding alone was insufficient. By the late twentieth century, the path forward had become clear: the field needed polymorphic markers, linkage maps, and population designs capable of converting polyploid complexity into interpretable recombination patterns.

## 3 Molecular linkage maps and first-generation QTL analysis

During 2000–2006, cotton entered the era of molecular linkage mapping. Zhang et al. (2002) constructed a molecular linkage map of allotetraploid cotton using a haploid population, establishing a framework for locating quantitative trait loci. With linkage maps, complex traits could begin to be analyzed as collections of genomic segments rather than as undifferentiated breeding values.

Early QTL studies were particularly influential when they identified loci with relatively large effects. Zhang et al. (2003) molecularly tagged a major QTL for fiber strength, demonstrating that a complex, economically important trait could be anchored to a genomic interval suitable for follow-up analysis. Additional QTL studies of fiber-related traits reinforced the view that lint yield and fiber quality were not only highly polygenic but also partly structured into genomic intervals that could be mapped and, eventually, combined in breeding programs (Mei et al., 2004).

Marker systems also became denser and more biologically informative during this period. Park et al. (2005) developed EST-derived microsatellites, providing an early bridge between genetic mapping and expressed genes, an important advance in cotton, where fiber traits arise from stage-specific transcriptional programs. As a result, mapping intervals could increasingly be linked to candidate pathways involving cell wall biosynthesis, cytoskeletal organization, and sugar transport, rather than remaining anonymous coordinates.

At the same time, these first-generation maps exposed a defining limitation of cotton genetics: QTL instability across environments. Many loci were conditionally expressed, and similar phenotypes could arise through different physiological pathways under different field conditions. The next phase, therefore, required higher marker density, broader multi-environment replication, and study designs capable of distinguishing robust QTL from environmentally fragile signals.

## 4 Multi-environment mapping and meta-analysis

To address QTL instability, cotton genetics increasingly integrated evidence across studies and environments during 2006–2012. Multi-environment QTL mapping for lint yield and fiber quality made reliability an explicit criterion: mapping was no longer concerned only with detection, but also with reproducibility under realistic production conditions (Yu et al., 2013).

Shen et al. (2006) mapped resistance to root-knot nematodes, illustrating a translational pattern that would become increasingly important in cotton: loci could be identified and then deployed to reduce pesticide dependence and stabilize yield. Disease- and pest-resistance loci were especially attractive because they often showed comparatively large effects and offered direct sustainability benefits.

Meta-QTL analysis emerged as a practical response to heterogeneity across studies. Said et al. (2013) synthesized QTL reports for fiber quality, yield, morphological traits, drought tolerance, and disease resistance, identifying consensus regions for prioritization. When individual studies lacked the power to define stable loci, aggregated evidence could reveal more robust signals.

Adaptation constraints also became more explicitly genetic during this period. Photoperiod dependence, a major barrier to expanding upland cotton into new latitudes, was addressed through induced mutation and subsequent genetic analysis, showing that key developmental switches could be reconfigured and mapped (Abdurakhmonov et al., 2007). This foreshadowed a broader lesson that remained important throughout later work: in cotton, mapping has the greatest translational value when it targets upstream regulators of phenology, disease resilience, and resource-use efficiency that can reshape downstream agronomic outcomes.

## 5 Linkage disequilibrium, association mapping, and population structure

During 2008–2017, cotton mapping expanded from biparental populations to broader germplasm panels, bringing population structure to the center of analysis. Cotton breeding has long been shaped by regional adaptation, market class, and intense selection for fiber standards; as a result, genetic relatedness is non-random, and linkage disequilibrium (LD) patterns vary across populations. Association mapping, therefore, required explicit treatment of both LD and population structure through careful measurement, modeling, and correction.

Cotton adopted the mixed-model framework widely used in crop association mapping, but its application in this species was constrained by features specific to allotetraploid genomes. Markers had to discriminate homoeologous loci, and inference had to remain robust despite uneven allele frequencies and subgenome-specific biases. Abdurakhmonov and Abdukarimov (2008) provided a broad synthesis of association-mapping frameworks for crop improvement, clarifying methodological principles relevant to cotton and other crops.

Early surveys of LD in both cultivated and exotic germplasm established the feasibility of association mapping in cotton by defining the population structure and LD-decay patterns that would guide later study design (Abdurakhmonov et al., 2008, Abdurakhmonov et al., 2009). Within this transition, association mapping of fiber-quality traits in upland cotton germplasm showed that LD-informed models could convert natural variation into interpretable marker–trait relationships with translational value for breeding (Abdurakhmonov et al., 2009). Similar analyses in *Gossypium barbadense* germplasm further demonstrated the utility of panel-based inference in a species where extra-long-staple fiber quality and adaptation are strongly shaped by population history (Abdullaev et al., 2017). Across these studies, a practical principle emerged: discoveries made in diverse germplasm are most useful when supported by replication and targeted validation.

## 6 SNP arrays and GWAS at scale

If association mapping was the conceptual shift, high-density SNP arrays were the engineering shift that made the approach scalable during 2015–2021. Hulse-Kemp et al. (2015) developed a 63K SNP array that provided standardized, high-throughput genotyping, effectively creating a common yarn that could be woven into comparable results across labs and breeding programs. This standardization changed cotton mapping from an artisanal craft into a cumulative science: loci discovered in one program could be interrogated in another without rebuilding the entire genotyping apparatus.

GWAS studies using dense marker maps began to identify favorable SNP alleles and candidate genes for traits related to fiber, yield, and other agronomic outcomes (Su et al., 2016). Cotton GWAS also expanded to address how population structure relates to agronomic trait architecture at broader scales, linking genotype to trait distributions across breeding contexts (Huang et al., 2017) and identifying genetic variation and candidate genes for important traits in structured panels (Sun et al., 2017).

Further platform development, including an 80K SNP array (Cai et al., 2017), strengthened the standardization ecosystem and made large-scale studies more routine. The cumulative effect was a new kind of resolution: rather than producing isolated QTL maps, cotton genetics began producing reusable genotype–phenotype maps that could be compared, meta-analyzed, and prioritized.

Multi-parent resources added another dimension during this era. Huang et al. (2021) developed an 8-way Upland cotton MAGIC population, creating recombination-rich panels that bridge linkage and association mapping. These resources accelerate discovery in breeding-relevant backgrounds by combining the diversity of multiple founders with controlled recombination.

## 7 Genomes add a new dimension: polyploid assemblies and regulatory mapping

Reference genomes transformed the meaning of cotton genomic coordinates during 2012–2021. The sequencing of allotetraploid cotton (TM-1) by Zhang et al. (2015) provided a scaffold for converting mapped loci into candidate genes and mechanistic hypotheses, ushering in a new phase of polyploid crop genetics. Once reference assemblies became available, QTL and GWAS signals could be interpreted in the context of gene models, synteny, and biological pathways, enabling stronger links between fiber traits and the underlying processes of cell wall formation, carbohydrate partitioning, and stress response.

Genome-scale resources also clarified that the two cotton subgenomes did not contribute equally to domestication and improvement. Wang et al. (2017a) presented evidence for asymmetric subgenome selection and cis-regulatory divergence, providing a mechanistic explanation for why some loci show greater consistency across mapping contexts: recurrent selection has shaped both regulatory architecture and coding sequence. In fiber biology, this distinction was especially important because many quality traits depend on coordinated transcriptional programs and developmental timing rather than on variation in a single structural gene.

Transcriptomic resources added another layer of interpretation. Zhang et al. (2013a) analyzed the *Gossypium arboreum* transcriptome under water stress, demonstrating that sequencing-based approaches can reveal candidate pathways and temporal dynamics. In cotton, however, expression resources were most informative when integrated directly with genetic mapping.

This integration emerged through genetical genomics and eQTL mapping in developing cotton fibers. Claverie et al. (2012) used cDNA-AFLP-based approaches to link genotype to expression variation during fiber development, enabling candidate-gene nomination by colocalizing trait and regulatory loci. Interspecific resources, including recombinant inbred line populations derived from *G. hirsutum* × *G. barbadense* crosses (Lacape et al., 2009), provided essential infrastructure for this integrative work by supplying the recombination and polymorphism needed to map both phenotypes and expression traits. The key genomic resources that enabled these advances are summarized in **Table 2**; **Supplementary Table 1**.

**Table 2:** Key genomic resources for cotton mapping.

Resource	What it enables	Polyploid-specific notes	Key references
Interspecific mapping populations (hirsutum × barbadense RILs)	QTL discovery + map-based cloning	High polymorphism; homoeolog resolution required	Lacape et al., 2009
SSR/EST marker systems	Early high-utility linkage maps	Transferable but limited density	Zhang et al., 2002; Park et al., 2005
SNP genotyping arrays (63K, 80K)	Standardized high-density genotyping	Subgenome specificity matters	Hulse-Kemp et al., 2015; Cai et al., 2017
GBS/BSA-seq mapping	Dense maps; rapid fine mapping	Needs homoeolog-aware calling	Zhao et al., 2017, Zhao et al., 2021
Reference genome (TM-1) and comparative assemblies	Candidate genes; synteny	Annotation and subgenome bias	Zhang et al., 2015
eQTL/genetical genomics in fiber	Regulatory mapping; causal nomination	Stage-specific programs; cis/trans logic	Claverie et al., 2012
SV-based pan-genomes and graph pan-genomes	SV-aware association; reduces reference bias	PAV and rearrangements become first-class variants	Jin et al., 2023; Zhang et al., 2026; Yang et al., 2026

## 8 From loci to cultivars: fine mapping, validation, and deployment

As marker density and population resources improved, cotton fine-mapping studies increasingly combined dense recombination analysis with transcript profiling to move from broad intervals to prioritized candidate genes. Liu et al. (2016) delimited a major multi-trait fiber QTL, the classical T1 region, to a narrow genetic interval and used RNA-seq and RT-PCR to prioritize likely causal genes. This study illustrated how expression data can strengthen the connection between statistical association and biological mechanism. Related work by Xu et al. (2017) localized introgressed fiber-length loci, including qFL-chr1, to sub-centimorgan intervals using near-isogenic backgrounds and substitution mapping, clarifying candidate pathways involving hormone signaling and transport processes while generating markers suitable for deployment. Similar studies integrating differential gene expression with QTL mapping for yield further emphasized that agronomic phenotypes reflect dynamic developmental processes and that mapping becomes more informative when intermediate biological layers are considered (Liu et al., 2011).

Disease resistance provided a direct bridge between genetic mapping and sustainability. Wang et al. (2009) mapped Fusarium wilt resistance loci, illustrating how genetic information can reduce dependence on chemical control and increase yield stability, particularly when validated and pyramided. Later work strengthened this translational pathway in two complementary ways. First, chromosome-scale localization by Jiang et al. (2009) showed that resistance could be conferred by multiple linked factors, with Verticillium wilt resistance QTL clustering on D-subgenome chromosomes D7 and D9. Second, LD-aware strategies combined association evidence with localized recombination to resolve clustered QTL into major and minor components, narrowing intervals and identifying candidate defense genes (Zhao et al., 2017).

At the population scale, GWAS in diverse upland cotton panels identified repeatable loci for resistance to Verticillium wilt and Fusarium wilt, including race-specific contexts, helping breeders prioritize markers with more consistent effects across genetic backgrounds and trials (Abdelraheem et al., 2020). Integrative pipelines that combined GWAS with QTL-seq and transcriptome sequencing further translated these signals into breeder-ready assays, including Kompetitive Allele-Specific PCR (KASP) markers for Verticillium resistance that can be used in routine selection (Zhao et al., 2021). Transcriptome profiling of *G. barbadense* under Verticillium challenge complemented this mapping framework by identifying responsive pathways and candidate mechanisms that could be aligned with mapped loci (Zhang et al., 2013b).

Studies of domestication and geographic adaptation added another dimension to translation by linking genome-scale selection patterns with classical trait dissection across the transition from wild to cultivated cotton (Grover et al., 2020). Understanding the genomic basis of differentiation and fiber improvement helped connect historical selection with modern breeding priorities and provided a framework for designing future crosses and introgressions (He et al., 2021). In parallel, trait mapping extended to architectural and phenological determinants of yield potential, such as the node of the first fruiting branch, reflecting a more mechanistic understanding of how development shapes productivity (Zhang et al., 2021).

Cotton now also provides examples of mapping-derived markers translated into practical selection. Darmanov et al. (2022) showed that marker-assisted selection can deliver superior fiber-quality cultivars in the Ravnaq series, providing a concrete example of how mapping moves from publication to field deployment (see **Box 1**). More broadly, syntheses of molecular markers and cotton genetic improvement have emphasized that future sustainability will depend on integrating mapping with predictive tools and realistic breeding workflows (Malik et al., 2014). **Table 3** catalogs functionally validated genes identified through cotton mapping studies, illustrating the field’s progression from statistical association to biological understanding; a more comprehensive list is provided in **Supplementary Table 2**.

**Table 3:** Functionally validated genes from cotton mapping studies.

Gene	Trait	Mapping approach	Validation method	Key finding	Reference
GhSAD1	Cold tolerance	GWAS (200 accessions)	VIGS; Arabidopsis OE	Regulates ABA signaling; HapB increases cold tolerance	Ge et al., 2022
GhCHS	Fiber elongation	eQTL mapping	RNAi in cotton	Flavonoid pathway; ROS accumulation affects elongation	Chen et al., 2026
GhDFR	Secondary wall synthesis	eQTL mapping	RNAi in cotton	Flavonoid pathway; cellulose biosynthesis	Chen et al., 2026
GhPIN3	Plant height	QTL mapping (BIL population)	VIGS	Auxin efflux carrier; silencing increases height	Ma et al., 2019
GhPAP	Fiber strength	SLAF-BSA-seq + InDel analysis	VIGS; Arabidopsis OE	Plastid lipid-associated protein; affects helix formation	Zhang et al., 2025a
GhABH	Fiber quality (multi-effect)	Fine mapping + KASP	VIGS	α/β-hydrolase; regulates cell wall thickness	Zhang et al., 2025b
GhTPS11	Flowering time	QTL mapping + transcriptome	VIGS; Arabidopsis OE	Trehalose-6-phosphate synthase; age pathway	Feng et al., 2026
GhE6	First fruiting branch height	RTM-GWAS + meta-QTL	VIGS	Early flowering regulation	Su et al., 2024
GhUBP15	Plant height	UAV-based GWAS	VIGS	Ubiquitin protease; plant architecture	Ye et al., 2023
GhCUL1	Plant height	UAV-based GWAS	VIGS	Cullin protein; plant architecture	Ye et al., 2023
GhCPR30	Verticillium wilt resistance	QTL mapping + BSA-seq	VIGS	Disease resistance; silencing increases susceptibility	Wang et al., 2023
miR477b	Fiber length	QTL co-localization	VIGS	Regulates DELLA via HOX3	Song et al., 2024
GbCYP72A1	Verticillium wilt resistance	QTL mapping	VIGS	Cytochrome P450; hormone signaling	Xu et al., 2023
GhALDH7B4	Fiber strength	SLAF-BSA-seq	VIGS; Arabidopsis OE	Aldehyde dehydrogenase; cell wall components	Tang et al., 2024
GhRBB1_A07	Fiber quality	MAGIC GWAS	Gene expression + SNP	Regeneration of bulb biogenesis; superior fiber	Islam et al., 2016

VIGS, virus-induced gene silencing; OE, overexpression; BIL, backcross inbred line; BSA-seq, bulk segregant analysis sequencing; GWAS, genome-wide association study; UAV, unmanned aerial vehicle; RTM-GWAS, restricted two-stage multi-locus GWAS.

Box 1 **Case study: the Ravnaq cultivars as a marker-to-deployment pipeline in cotton.**

The Ravnaq cultivar series provides an instructive example of long-horizon translation from LD-informed discovery to cultivar release in cotton. The translation pipeline unfolded over approximately 15 years:

**Table:** 

Phase	Activities	Key outputs	Duration	References
LD characterization	Survey of LD patterns in 335 *G. hirsutum* accessions; population structure analysis; identification of LD blocks associated with fiber quality	First comprehensive LD map in cotton; established the feasibility of association mapping in cotton germplasm	2005-2009	Abdurakhmonov et al., 2008, Abdurakhmonov et al., 2009
Marker-trait association	Multi-environment phenotyping (Uzbekistan, Mexico); MLM-based association mapping with Q+K correction	20–25 SSR markers associated with fiber length, strength, and micronaire across environments	2008-2012	Abdurakhmonov et al., 2008, Abdurakhmonov et al., 2009
Validation and refinement	Cross-validation in independent panels; QTL co-localization; meta-analysis	Confirmed stable associations; narrowed candidate intervals	2010-2014	Abdullaev et al., 2017
Marker conversion	Development of breeder-friendly PCR assays; conversion of associated SSRs to high-throughput formats	Deployable markers for MAS	2012-2015	Darmanov et al., 2022; Kushanov et al., 2016
Marker-assisted backcrossing	Five generations of backcrossing with foreground selection (BC_5_); background recovery using genome-wide markers	Near-isogenic lines with introgression of favorable LD blocks	2014-2019	Darmanov et al., 2022
Multi-location trials	Testing across diverse environments in Uzbekistan: yield and fiber quality assessment	Consistent superiority in fiber strength (38.3 vs 32.5 g tex^-1^) and length (1.18 vs 1.12 inch)	2017-2021	Darmanov et al., 2022
Cultivar release	Distinctness, uniformity, stability testing; certification	‘Ravnaq-1’ and ‘Ravnaq-2’ released for commercial cultivation	2021-2022	Darmanov et al., 2022

Several factors underpinned this translational success. Long-term institutional commitment over more than 15 years provided the continuity needed to sustain a complex marker-to-cultivar pipeline. Equally important was the close integration of discovery research with applied breeding, which enabled fundamental findings to be rapidly tested in breeding-relevant genetic backgrounds. Early investment in linkage disequilibrium characterization, undertaken before GWAS became routine, established a foundation for understanding population structure and marker–trait relationships that informed subsequent stages of the program (Abdurakhmonov et al., 2008, Abdurakhmonov et al., 2009). The validation strategy combined the breadth of diversity panels with the precision of structured mapping populations, using both approaches to confirm associations before marker deployment (Kushanov et al., 2016). Associated SSR markers were then converted into breeder-friendly, higher-throughput formats suitable for routine screening in applied breeding programs.

Sustained international collaboration, particularly the Uzbekistan–USDA partnership, further strengthened this effort by combining complementary expertise, environments, and breeding resources (Darmanov et al., 2022). Importantly, trait–marker associations were evaluated across three distinct environments, Uzbekistan, the United States (College Station, Texas), and Mexico (USDA-ARS Winter Nursery), which helped identify the most stable associations across contrasting production settings (Abdurakhmonov et al., 2008, Abdurakhmonov et al., 2009). Collectively, these features show that successful translation in cotton genetics depends not only on robust science but also on long-time horizons, coordinated validation, and sustained collaboration across the discovery-to-deployment continuum. More broadly, this case suggests that, in a polyploid crop with modest polymorphism, the transition from LD-based discovery to cultivar release is feasible but requires sustained integration across the discovery, validation, and breeding phases.

## 9 Structural variation and graph pan-genomes: beyond SNP-centric mapping

From 2023 onward, cotton mapping has increasingly moved beyond SNP-centric, single-reference frameworks based primarily on biallelic variation. Cotton increasingly challenges these assumptions because structural variation, presence/absence variation, and genomic rearrangements, especially across wild, landrace, and geographically diverse germplasm, can have large functional effects while remaining invisible to SNP-only approaches.

Jin et al. (2023) provided direct evidence for this shift by combining an SV-based cotton pan-genome with GWAS. Their study showed that structural variants are not merely genomic curiosities, but trait-associated variants that can reshape mapping outcomes and alter which alleles are realistically deployable in breeding. This marked a qualitative change in the questions cotton genetics could address. Instead of asking only which SNP tags a QTL, researchers could begin to ask which structural haplotype is likely causal and how to track it in breeding populations.

Graph-based genomes represent the next major advance in this framework because they enable more comprehensive incorporation of structural variation into discovery and breeding pipelines. A newly published complementary study by Zhang et al. (2026) further sharpened this transition by generating a telomere-to-telomere (T2T) genome for the elite cultivar NDM13 together with near-T2T assemblies for 27 additional representative Gossypium hirsutum accessions. Using these resources, along with transcriptomic profiling across 15 tissues and population-scale analyses, the authors constructed a T2T-reference-based pangenome and identified 761,536 structural variants across 1,671 worldwide accessions evaluated in 22 environments, linking previously hidden SVs with breeding-relevant traits. Together with the pan-genome graph study by Yang et al. (2026), which used 107 assemblies spanning the wild-to-domesticated continuum and resolved six large-scale structural variants, these results show that modern cotton mapping is moving toward complementary T2T-reference, population-scale SV, and graph-based frameworks. For cotton improvement, the implication is immediate: some of the most useful alleles may not be fully represented or genotyped in conventional linear-reference, SNP-centric pipelines, and future mapping efforts will increasingly need to capture structural haplotypes, copy-number differences, and presence/absence variation directly.

Recent work has also expanded cotton mapping beyond sequence variation alone. Huang et al. (2024) integrated epigenomic and three-dimensional genomic analyses, revealing developmental dynamics and subgenome asymmetry in the transcriptional regulatory architecture of allotetraploid cotton. This study showed that mapping now extends beyond DNA sequence polymorphism to include chromatin conformation and regulatory topology, adding new mechanistic dimensions to the interpretation of complex traits.

Further comparative genomics in elite cotton lines further strengthened this point. Sreedasyam et al. (2024) generated high-quality genomes for three modern cultivars. They updated TM-1, showing that substantial sequence and structural variation persists even among modern breeding materials and overlaps with transcriptional differences relevant to fiber development. Together with graph-based population resources, these results show that SV analysis becomes more informative when paired with expression and other functional datasets, because the practical question is not only where an SV occurs but whether it changes gene activity in ways that matter for breeding.

## 10 Deployment pipelines for sustainability: how mapping reduces inputs, not only time

Cotton mapping delivers its greatest practical value not simply by identifying loci, but by reducing the economic and environmental costs of crop improvement. Its contribution to sustainability lies in reducing breeding trial burden, enabling the assembly of multiple favorable traits, and improving performance stability under stress.

Disease-resistance loci provide some of the clearest examples. Once mapped, resistance loci can be pyramided to reduce dependence on pesticides and improve yield stability (Wang et al., 2009; Jiang et al., 2009; Zhao et al., 2017; Abdelraheem et al., 2020; Zhao et al., 2021). Abiotic-resilience traits become equally important sustainability targets when mapping enables selection for tolerance that stabilizes productivity under drought, heat, or salinity without increasing input demand. Abdelraheem et al. (2017) provided a comprehensive meta-analysis of QTL associated with abiotic and biotic stress resistance in tetraploid cotton, synthesizing decades of discovery into a more actionable framework for breeding.

Fiber-quality improvement represents a distinct sustainability pathway. Greater fiber quality and improved stability increase value per hectare and reduce waste across the production and textile value chain. Wild introgression studies that identify fiber-quality QTL alleles from *Gossypium tomentosum* (Zhang et al., 2011) show how biodiversity can be reintroduced into elite breeding when mapped loci guide the recovery of agronomically suitable backgrounds. In this sense, sustainability is not limited to input reduction alone, but also includes improving output value and resource-use efficiency. **Table 4** summarizes how mapping has contributed to sustainability across major trait domains; a more detailed version is provided in **Supplementary Table 3**.

**Table 4:** Trait exemplars: where mapping most clearly touches sustainability (abridged version; full table in **Supplementary Table 3**).

Trait domain	Typical targets	What made it hard	Mapping-to-mechanism bridge	Breeding leverage (sustainability link)	Key references
Fiber quality	QTL clusters; pleiotropy; stage-specific control; allele discovery via GWAS	Single-cell development; strong G×E; homoeolog redundancy	Fine-mapping + expression support; eQTL co-localization; SV-aware scans	Higher value per hectare; quality stability reduces waste and energy in processing	Mei et al., 2004; Abdurakhmonov et al., 2009; Sun et al., 2017; Liu et al., 2016; Xu et al., 2017; Zhang et al., 2025a, Zhang et al., 2025b; Tang et al., 2024; Islam et al., 2016
Lint yield and stability	Polygenic loci; environment-responsive QTL; yield-component dissection	Strong field heterogeneity; correlated traits; management effects	Multi-environment QTL + transcriptome-linked loci	Stable yield reduces land expansion pressure and input volatility	Yu et al., 2013; Liu et al., 2011
Earliness/phenology and architecture	Maturity SNPs; fruiting branch traits; flowering/earliness loci	Trade-offs with yield/fiber; photoperiod sensitivity; breeding-region stratification	GWAS + candidate genes; QTL-seq for key architectural traits	Earlier harvest windows reduce risk, irrigation needs, and pest pressure	Su et al., 2016; Huang et al., 2017; Zhang et al., 2021; Abdurakhmonov et al., 2007; Ma et al., 2019; Feng et al., 2026; Su et al., 2024
Verticillium wilt resistance	Major-effect loci plus quantitative background; resistance QTL clusters	Pathogen diversity; durability challenge; environment interactions	QTL clustering + fine mapping; integrative GWAS/QTL-seq/transcriptome → KASP markers	Durable resistance reduces fungicide use and yield losses	Jiang et al., 2009; Zhao et al., 2017; Abdelraheem et al., 2020; Zhao et al., 2021; Wang et al., 2023; Xu et al., 2023
Fusarium wilt resistance	Major resistance genes/loci; panel-wide QTL	Race structure and geographic spread	Gene mapping + GWAS confirmation in breeding germplasm	Reduced chemical inputs and replanting; resilience in infested soils	Wang et al., 2009; Abdelraheem et al., 2020
Nematode resistance	Resistance QTL for root-knot nematodes	Phenotyping is destructive and environment-dependent	Interval/QTL mapping with validated markers	Reduced nematicide reliance; safer soils and water	Shen et al., 2006
Abiotic resilience	Meta-QTL for tolerance; expression-responsive loci	Strong G×E; drought timing effects; complex physiology	Meta-QTL synthesis; stress transcriptomes; eQTL and prediction	Water productivity and yield stability under climate volatility	Said et al., 2013; Zhang et al., 2013a; Abdelraheem et al., 2017; Ge et al., 2022
Domestication and geographic differentiation	Selection sweeps; cis-regulatory divergence; improvement loci	Bottlenecks; subgenome asymmetry; introgression vs local adaptation	Population genomics + regulatory divergence; mapping of improvement signals	Broadens adaptive diversity; guides pre-breeding for resilience	Wang et al., 2017a; He et al., 2021; Grover et al., 2020
Pan-genomes, SV, and graph coordinates	SV-based GWAS; graph pan-genomes; structural haplotypes	Reference bias; SV genotyping; subgenome-aware coordinates	SV-aware association on pan-genomes; epigenome/3D-genome context	Captures hidden diversity and reduces false negatives; enables durable stacking	Jin et al., 2023; Zhang et al., 2026; Yang et al., 2026; Huang et al., 2024
Multi-parent resources (MAGIC)	Allele-series mapping; recombination-rich panels	Construction cost; phenotyping scale	MAGIC, GWAS, bridging linkage and association	Accelerates discovery and validation in breeding-relevant backgrounds	Huang et al., 2021

Taken together, these studies show that cotton mapping now supports a more environmentally smart breeding model, in which selection is guided not only by yield potential but also by stability under stress, disease pressure, and resource limitations. In this way, cotton genetic mapping functions increasingly as an applied plant biotechnology platform for sustainable crop improvement.

## 11 Cotton’s journey comparisons with other crops

Cotton’s mapping trajectory is clearer when compared with other major crops. In maize, rapid decay of linkage disequilibrium enables high-resolution mapping but requires very dense marker coverage (Romay et al., 2013). By contrast, cotton’s predominantly self-pollinating habit and domestication bottlenecks produced broader LD blocks, which facilitated early QTL discovery with sparse markers but later required larger populations and stricter statistical control for fine mapping and association analysis (Abdurakhmonov et al., 2009; Abdurakhmonov and Abdukarimov, 2008).

Rice benefited from the earlier availability of a complete reference genome, which accelerated the conversion of QTL intervals into candidate genes and provided useful models for multiparent population design and stress mapping (International Rice Genome Sequencing Project, 2005; Feng et al., 2016; Huang et al., 2021). Wheat offers a different polyploid comparison: although its larger, more repetitive genome initially slowed progress, international genomic resources helped accelerate discovery, and the integration of GWAS with genomic selection for quality traits provides a strategy increasingly relevant to cotton improvement (International Wheat Genome Sequencing Consortium, 2018).

Soybean presents a closer parallel in the structure of elite germplasm, but its greater ease of transformation has generally enabled faster functional validation of candidate genes. In cotton, by contrast, validation has depended more heavily on approaches such as virus-induced gene silencing and near-isogenic line analysis (Xu et al., 2017; Zhao et al., 2021; Ge et al., 2022). Model systems such as Arabidopsis have also provided methodological templates, particularly for regulatory mapping and for integrating eQTL analysis with functional validation.

What distinguishes cotton is that polyploidy repeatedly drove methodological innovation rather than simply complicating implementation. Homoeolog-aware marker development, subgenome-specific expression analysis, and, more recently, SV-based pan-genomes and graph-based frameworks were advanced in response to cotton’s distinctive genomic architecture. In this sense, cotton has not only adopted methods from other crops but has also helped push them toward greater analytical flexibility.

## 12 Gene regulatory networks: from single genes to systems

The transition from mapping loci to understanding gene function in cotton has increasingly shifted attention toward regulatory networks. Early eQTL studies in developing cotton fibers showed that expression variation for many genes mapped to trans-acting hotspots, indicating coordinated regulatory control during fiber development (Claverie et al., 2012; Liu et al., 2011). Chromosome 21 emerged as a notable hotspot for fiber-expressed genes, with multiple eQTLs colocalizing with phenotypic QTLs for fiber-quality traits (Claverie et al., 2012).

The integration of transcriptomics with QTL mapping has been especially powerful in cotton. Liu et al. (2016) combined fine mapping of the T1 fiber-quality QTL with RNA-seq to identify candidate genes whose expression differences between parental lines were associated with the phenotype. This strategy, in which expression data are used to prioritize positional candidates, has become an important component of post-GWAS validation in cotton (Xu et al., 2017; Zhao et al., 2021; Huang et al., 2024).

More recently, epigenomic and three-dimensional genomic analyses have shown that the two cotton subgenomes are not transcriptionally equivalent. Huang et al. (2024) reported that the Dt subgenome exhibits more dynamic chromatin accessibility during development than the At subgenome, offering one possible explanation for why many fiber-quality QTLs map to the D subgenome even though its diploid progenitor did not produce spinnable fibers (Jiang et al., 1998). This asymmetry also extends to regulatory-element distribution: cis-regulatory elements showing signatures of selection during domestication are enriched in the D subgenome (Huang et al., 2024; Song et al., 2024).

Small RNAs add a further layer of regulatory complexity. For example, miR477b, which colocalizes with a fiber-length QTL, exhibits differential expression between long- and short-fiber lines, and silencing experiments support its role in fiber elongation by regulating DELLA and downstream targets (Song et al., 2024). Similarly, flavonoid-pathway genes such as GhCHS and GhDFR, identified through eQTL mapping, appear to influence fiber elongation and secondary wall synthesis through distinct regulatory mechanisms (Chen et al., 2026).

Together, these systems-level findings show that cotton phenotypic variation arises not only from coding-sequence polymorphisms but also from regulatory variation distributed asymmetrically across subgenomes and modulated by small RNAs, chromatin accessibility, and three-dimensional genome architecture. Future advances in cotton mapping will therefore depend on methods that can capture this regulatory complexity more directly.

## 13 A cotton-native mapping pipeline: conceptual synthesis

**Figure 2:**
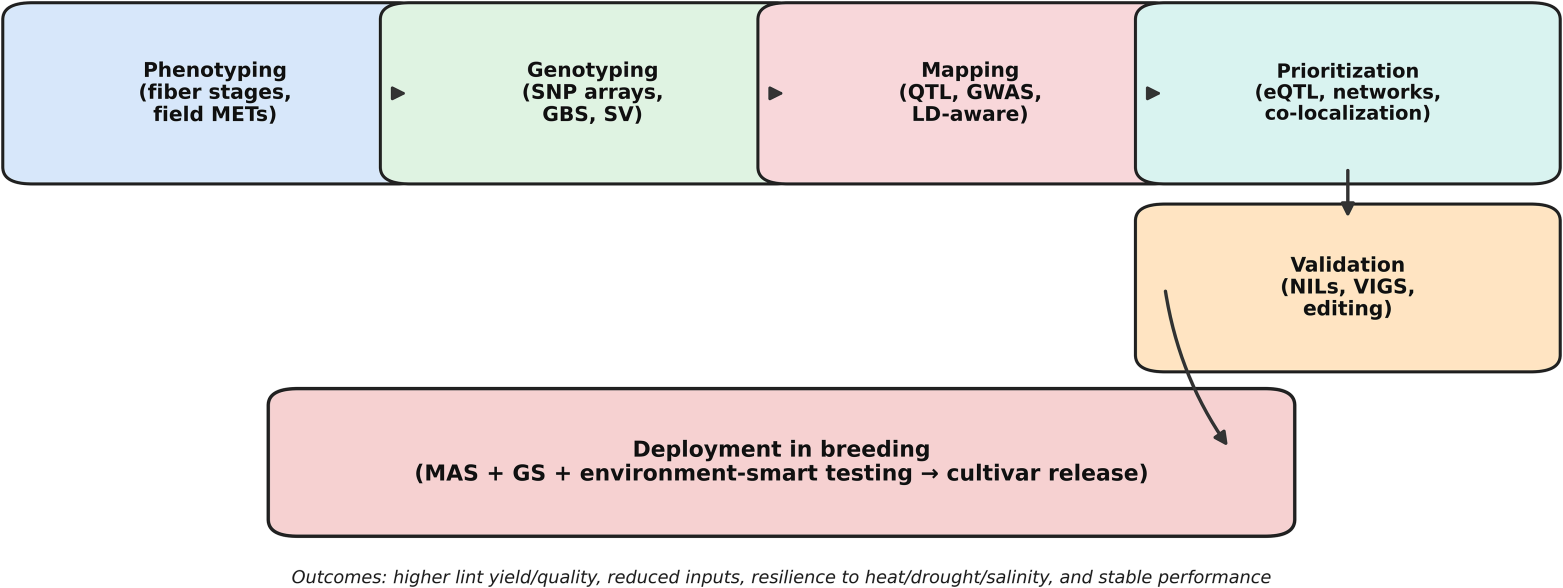
A cotton mapping-to-deployment pipeline. Durable translation requires phenotype design (including multi-environment trials), standardized genotyping (arrays/GBS/SV), LD-aware inference (QTL/GWAS), regulatory prioritization (eQTL and networks), and validation (NILs, VIGS, editing), culminating in deployment through MAS and emerging genomic prediction.

Cotton’s mapping trajectory increasingly resembles an integrated pipeline (**Figure 2**). Phenotyping remains the major source of cost and irreproducibility, whereas genotyping has become increasingly standardized. At the same time, inference has become more sensitive to linkage disequilibrium and population structure, and faster functional assays and genome-editing tools are improving the capacity for validation. A central lesson from recent work is that cotton mapping is most durable when it integrates three complementary forms of evidence: (1) genetic evidence, including stable QTL or GWAS signals supported by multi-environment replication; (2) regulatory evidence, including expression modules and eQTL colocalization that help prioritize candidate genes; and (3) direct validation, including near-isogenic backgrounds, virus-induced gene silencing, or CRISPR-based editing. This integrative pipeline provides the practical foundation through which cotton genetic mapping contributes to plant biotechnology.

## 14 Persistent challenges in cotton mapping

Despite remarkable progress, several challenges persist in cotton genetic mapping. Homoeolog discrimination remains non-trivial even with short-read sequencing; many SNPs initially identified as polymorphisms between parents prove to be homoeologous sequence variants rather than true allelic differences (Hulse-Kemp et al., 2015; Page et al., 2016). This requires careful validation, often through subgenome-specific PCR or long-read sequencing.

Phenotyping throughput for fiber traits remains a bottleneck. Unlike above-ground traits that can be scored by UAVs (Ye et al., 2023), fiber quality assessment requires destructive harvesting and laboratory analysis (HVI, AFIS). This limits population sizes and replication, reducing power to detect small-effect QTLs. The gap between genotyping (millions of markers) and phenotyping (hundreds of lines) continues to widen.

G×E complexity is particularly acute in cotton, grown across diverse latitudes, water regimes, and management systems. QTLs identified in one environment frequently fail to replicate elsewhere (Yu et al., 2013; Said et al., 2013). Multi-environment trials are essential but costly, and statistical models that accurately predict G×E remain underdeveloped.

Validation throughput lags discovery. While GWAS and QTL mapping routinely identify dozens of loci, functional validation (VIGS, transgenics, genome editing) remains labor-intensive and low-throughput. The gap between candidate genes and validated genes widens each year.

Structural variation is only beginning to be systematically captured at the levels required for breeding, despite rapid recent progress in SV-based pan-genomes, T2T-reference population studies, and graph pan-genomes (Jin et al., 2023; Zhang et al., 2026; Yang et al., 2026). Presence-absence variation, copy number variants, and inversions likely contribute substantially to trait variation but are missed by SNP-centric approaches. Graph-based pan-genomes address this but require computational infrastructure not yet routine in breeding programs.

While gene editing in cotton is advancing, it still faces challenges related to efficiency and genotype dependence. Translating validated genes into edited cultivars requires optimization for each target and background. These challenges define the research agenda ahead: better homoeolog-aware analysis tools, high-throughput phenomics, environment-aware prediction models, scalable validation platforms, and routine incorporation of structural variation.

## 15 A practical workflow for cotton GWAS and marker deployment

For applied cotton GWAS, study design should begin with a panel matched to the breeding objective: elite-only panels for within-program deployment, broader diversity panels for allele discovery, and multi-parent or introgression resources when recombination or exotic diversity is limiting. Phenotyping should span multiple years or representative target environments, with common checks and standardized fiber assays, because single-season associations are rarely sufficient in cotton given strong genotype-by-environment interaction and phenotyping noise (Abdurakhmonov et al., 2008, Abdurakhmonov et al., 2009; Yu et al., 2013; Said et al., 2013; Hulse-Kemp et al., 2015; Huang et al., 2021). Marker datasets should then be filtered in a polyploid-aware manner to remove ambiguously mapped loci, control missingness and allele frequency, and evaluate population structure, kinship, and LD decay before association testing.

Model choice should follow the breeding question rather than convention alone. Mixed-model GWAS remains appropriate for detecting interpretable loci. Still, post-GWAS prioritization should emphasize replication across environments, local LD support, biological plausibility, and convertibility into robust assays such as KASP, CAPS, or other breeder-friendly markers. Markers become breeder-usable only when their effects are sufficiently large, stable, and easy to score in routine germplasm; loci that are small, background-dependent, or highly environment-sensitive are better treated as inputs to multi-locus prediction than as stand-alone selection tools (Liu et al., 2016; Xu et al., 2017; Zhao et al., 2021; Darmanov et al., 2022).

In practice, cotton breeders therefore face a decision point after discovery. Marker-assisted selection is most realistic for major or moderately strong loci with repeated support across populations and trials, especially for disease resistance, phenology, or fiber loci that can be validated in breeding backgrounds. When many loci are individually weak, or when phenotyping is expensive and genotype-by-environment interaction is large, genomic selection or combined marker-plus-prediction strategies are often more appropriate than locus-by-locus deployment. In this sense, marker discovery and marker usability should be treated as connected but distinct stages: GWAS identifies candidate leverage points, whereas breeding pipelines require evidence that those points are stable, cost-effective, and biologically credible enough to influence routine selection decisions.

After five decades of cotton genetic mapping, several practical lessons emerge for investigators entering the field. An important early step is to characterize linkage disequilibrium in the target germplasm, because LD structure directly affects marker-density requirements, mapping resolution, and the interpretability of association signals. In cotton, even a modest initial investment in LD assessment can generate substantial downstream benefits for study design, locus prioritization, and validation (Abdurakhmonov et al., 2008, Abdurakhmonov et al., 2009).

Polyploidy should not be regarded as a complication to be filtered out, but rather as a defining feature of cotton biology. Homoeologous gene pairs often retain functional relevance, and subgenome-specific effects remain central to trait interpretation (Wang et al., 2017a; Claverie et al., 2012). In particular, the D subgenome repeatedly contributes loci associated with fiber-quality variation in cultivated cotton, despite the limited contribution of its diploid progenitor to spinnable fiber (Jiang et al., 1998; Mei et al., 2004).

A further lesson is that statistical associations should be treated as hypotheses rather than endpoints. Robust translation depends on validation across environments and on convergence among multiple lines of evidence, including genetic association, regulatory support, and functional testing (Abdelraheem et al., 2017; Liu et al., 2016; Darmanov et al., 2022). Long-lived community resources, such as recombinant inbred lines, MAGIC populations, NAM populations, and chromosome segment substitution lines, often repay their development costs by enabling older questions to be revisited as genomic tools improve (Huang et al., 2021; Lacape et al., 2009).

As genotyping has become cheaper and more routine, high-quality phenotyping has emerged as the principal rate-limiting step. Multi-environment trials are expensive but often indispensable for distinguishing durable effects from transient associations (Ye et al., 2023). Given the magnitude of genotype-by-environment interaction in cotton, loci that appear strong in one season or location may weaken or disappear in another, making repeated evaluation across years and environments essential for identifying stable, deployable effects (Yu et al., 2013; Said et al., 2013; Abdelraheem et al., 2017).

Wild relatives preserved in germplasm collections are also likely to harbor valuable alleles that remain underused in modern breeding pools (Zhang et al., 2011; Wang et al., 2012, Wang et al., 2016; Wang et al., 2017b; Chen et al., 2020). At the same time, movement from discovery to cultivar release typically requires long timelines, often a decade or more. It proceeds most effectively when discovery researchers, breeders, and deployment partners remain coordinated across the full pipeline (Darmanov et al., 2022). Progress will also accelerate when the international cotton community shares germplasm resources, markers, and both successes and negative results, thereby improving reproducibility, increasing comparability across studies, and reducing redundant effort.

Taken together, these lessons provide a practical foundation for the next phase of cotton mapping and for its continuing contribution to plant biotechnology.

## 16 Future directions for cotton genetic mapping

The next phase of cotton mapping is likely to be defined by three priorities: a richer representation of genomic variation, stronger validation frameworks, and closer integration of discovery, prediction, and deployment. First, graph-based genomes and SV-aware pan-genomes may become the standard framework for cotton discovery because they reduce reference bias and capture variation increasingly relevant to breeding (Jin et al., 2023; Zhang et al., 2026; Yang et al., 2026). Recent cotton studies clarify why this shift matters. Comparative assemblies of modern cotton lines revealed substantial sequence, structural, and gene-content variation even among elite cultivars, together with transcriptional differences linked to fiber development and likely trait divergence (Sreedasyam et al., 2024). More recently, complementary advances have appeared at two scales: Zhang et al. (2026) produced a T2T reference for elite cultivar NDM13 plus near-T2T assemblies for 27 additional upland cotton accessions and used these resources to identify 761,536 SVs across 1,671 accessions with phenotypes from 22 environments, whereas Yang et al. (2026) built a graph pan-genome from 107 assemblies across the wild-to-domesticated continuum and resolved six large-scale structural variations shaping evolutionary history and agronomic trait architecture. Together, these studies suggest that future cotton mapping will increasingly require integration of T2T assemblies, population-scale SV catalogs, graph coordinates, transcriptomics, regulatory data, and phenomics to prioritize structurally complex loci for breeding.

Second, cotton improvement will need to integrate mapping more closely with multi-environment prediction. Climate variability makes genotype-by-environment interaction an unavoidable component of trait architecture, so mapping results must increasingly be judged by their stability, transferability, and predictive value. Dense and standardized genotyping platforms are already available (Hulse-Kemp et al., 2015), but harmonized phenotyping and environment-aware models will determine whether predictive breeding becomes routine at scale.

Third, validation will determine which discoveries prove durable. In a polyploid crop, causal inference remains difficult without direct testing because of homoeolog redundancy and regulatory compensation, which can mask functional effects. Genomic analyses of subgenome divergence further underscore the need for validation strategies that are themselves homoeolog-aware (Wang et al., 2017a).

Beyond sequence variation alone, epigenomic and three-dimensional genomic context will increasingly inform mapping. The work of Huang et al. (2024) shows that regulatory architecture varies across developmental stages and between subgenomes, and that this variation will be essential for understanding how alleles function within their biological context.

Several technical bottlenecks will determine how quickly these advances become usable in breeding. SV discovery still varies across sequencing platforms, read lengths, callers, and graph-construction strategies, making cross-study comparisons and coordinate transfer difficult. Pan-genome resources also lack fully standardized conventions for graph construction, SV representation, and benchmark truth sets. At the same time, multi-omics integration remains limited by tissue specificity, developmental timing, and uneven sampling across environments. Practical breeding use adds another layer: most programs still need low-cost ways to genotype priority SVs, impute them accurately from short reads, and convert them into portable assays for routine screening. Near-term progress will therefore depend on harmonized SV standards, shared benchmark panels, graph-aware imputation pipelines, and marker-conversion workflows that reduce complex haplotypes to breeder-usable tests.

More broadly, cotton’s history supports an important conclusion: technically difficult crops often drive methodological progress. Across successive eras, each major challenge generated tools and analytical frameworks that later proved useful beyond cotton itself. The long-term outcome, cultivars that are more productive, resilient, and resource-efficient, will depend on sustained scientific cooperation and on the effective integration of genomic discovery with breeding application.

## Supplementary material

Supplementary Table 1 Additional genomic resources for cotton mapping (expanded from **Table 2**).

Supplementary Table 2 Additional functionally validated genes from cotton mapping studies (expanded from **Table 3**). VIGS, virus-induced gene silencing; OE, overexpression; BIL, backcross inbred line; BSA-seq, bulk segregant analysis sequencing; GWAS, genome-wide association study; UAV, unmanned aerial vehicle; RTM-GWAS, restricted two-stage multi-locus GWAS; CSSL, chromosome segment substitution line; LP, lint percentage; FL, fiber length; ROS, reactive oxygen species; TF, transcription factor.

Supplementary Table 3 Complete trait exemplars: where mapping most clearly touches sustainability (expanded from **Table 4**).
