# Supplementary material for: Cotton genetic mapping for plant biotechnology: from markers to graph pan-genomes and sustainable breeding
Source: Front Plant Sci. 2026 May 12;17:1825852. doi: 10.3389/fpls.2026.1825852 (PMC13201509; doi:10.3389/fpls.2026.1825852)
Supplement: Supplementary Table 1 — Additional genomic resources for cotton mapping (expanded from Table 2). [file Table1.docx]

**Supplementary Table S1. Additional genomic resources for cotton mapping (expanded from Table 2)**

| **Resource** | **Description** | **What It Enables** | **Polyploid-Specific Notes** | **Key References** |
| --- | --- | --- | --- | --- |
| Interspecific mapping populations (hirsutum × barbadense RILs) | Recombinant inbred lines from crosses between cultivated species | QTL discovery; map-based cloning; genetic resolution | High polymorphism; requires homoeolog resolution | Lacape et al., 2009 |
| SSR/EST marker systems | Microsatellite markers from genomic and expressed sequences | Early linkage maps; candidate gene tagging | Transferable but limited density | Zhang et al., 2002; Park et al., 2005 |
| SNP genotyping arrays (63K, 80K) | High-density SNP chips for standardized genotyping | Genome-wide association studies; genomic selection | Subgenome specificity requires careful design | Hulse-Kemp et al., 2015; Cai et al., 2017 |
| GBS/BSA-seq mapping | Genotyping-by-sequencing; bulk segregant analysis | Rapid fine mapping; QTL-seq | Needs homoeolog-aware variant calling | Zhao et al., 2017; Zhao et al., 2021 |
| Reference genome (TM-1) and comparative assemblies | Whole genome sequence of *G. hirsutum* and related species | Candidate gene identification; synteny analysis | Annotation and subgenome bias require attention | Zhang et al., 2015; Wang et al., 2017a |
| eQTL/genetical genomics in fiber | Expression QTL mapping in developing fibers | Regulatory mapping; causal nomination | Stage-specific programs; cis/trans logic | Claverie et al., 2012 |
| SV-based pan-genomes and graph pan-genomes | Structural variation-aware genome representations | SV-aware association; reduced reference bias | PAV and rearrangements become first-class variants | Jin et al., 2023; Zhang et al., 2026; Yang et al., 2026 |
| Chromosome segment substitution lines (CSSLs) | Lines carrying defined chromosome segments from wild donors | Fine mapping; introgression of exotic alleles | Enables the dissection of individual chromosome segments | Wang et al., 2016; Chen et al., 2020 |
| Chromosome substitution lines | Lines with entire chromosomes substituted from wild species | Chromosome-specific effect analysis; gene mapping | Powerful for dissecting inter-genomic interactions | Saha et al., 2013 |
| Wild introgression populations | Populations derived from crosses with wild relatives | Allele mining; diversity expansion | Captures alleles absent from elite germplasm | Zhang et al., 2011; Wang et al., 2012; Wang et al., 2017b |
| MAGIC populations | Multi-parent advanced generation inter-cross populations | High recombination; fine resolution; balanced allele frequencies | Complex development; sophisticated analysis required | Huang et al., 2021 |
| NAM populations | Nested association mapping populations | High power; controlled relatedness; broad allele sampling | Resource-intensive; limited to founder alleles | Chidzanga et al., 2022 |
| BAC libraries | Bacterial artificial chromosome libraries | Physical mapping; map-based cloning | Essential for large genome assembly | Wang et al., 2007; Hu et al., 2009 |
| Fiber transcriptome atlases | Comprehensive expression data across fiber development | Gene discovery; regulatory network analysis | Stage-specific; subgenome resolution | Zhang et al., 2013a; Claverie et al., 2012 |
| Small RNA and degradome data | Small RNA expression and target identification | Regulatory network analysis; miRNA discovery | Additional layer of regulatory complexity | Song et al., 2024; Cai et al., 2021 |
| Epigenomic and 3D genomic maps | Chromatin accessibility; methylation; 3D conformation | Regulatory architecture; cis-element discovery | Reveals subgenome asymmetry; developmental dynamics | Huang et al., 2024 |
| Mutant populations | EMS-induced mutant libraries | Reverse genetics; functional validation | Resource for gene function studies | Patel et al., 2022 |
| VIGS vectors | Virus-induced gene silencing systems | Rapid functional validation in cotton | Bypasses stable transformation requirement | Various |
| CRISPR-Cas9 systems | Genome editing platforms | Gene function validation; trait improvement | Efficiency and genotype dependency remain challenges | Various |
| CAPS/dCAPS markers | Cleaved amplified polymorphic sequence markers | Gene-specific marker development | Convert association signals to breeder-ready assays | Kushanov et al., 2016 |
| KASP markers | Kompetitive allele-specific PCR markers | High-throughput genotyping; breeder-friendly | Scalable; cost-effective for MAS | Zhao et al., 2021 |
| Consensus genetic maps | Integrated maps combining multiple populations | Comparative mapping; meta-analysis | Provides framework for QTL integration | Blenda et al., 2012; Yu et al., 2010 |
| CottonQTLdb | Database of published QTLs | Meta-analysis; QTL prioritization | Community resource for marker selection | Said et al., 2015 |
| CottonGen database | Comprehensive genomic and genetic data repository | Data sharing; community resource | Essential for collaborative progress | Yu et al., 2014 |
| High-throughput phenotyping platforms | UAV-based; spectral imaging; automated measurement | Enhanced phenotyping capacity; temporal data | Addresses phenotyping bottleneck | Ye et al., 2023 |
