# Supplementary material for: Cotton genetic mapping for plant biotechnology: from markers to graph pan-genomes and sustainable breeding
Source: Front Plant Sci. 2026 May 12;17:1825852. doi: 10.3389/fpls.2026.1825852 (PMC13201509; doi:10.3389/fpls.2026.1825852)
Supplement: Supplementary Table 2 — Additional functionally validated genes from cotton mapping studies (expanded from Table 3). VIGS, virus-induced gene silencing; OE, overexpression; BIL, backcross inbred line; BSA-seq, bulk segregant analysis sequencing; GWAS, genome-wide association study; UAV, unmanned aerial vehicle; RTM-GWAS, restricted two-stage multi-locus GWAS; CSSL, chromosome segment substitution line; LP, lint percentage; FL, fiber length; ROS, reactive oxygen species; TF, transcription factor. [file Table2.docx]

**Supplementary Table S2. Additional functionally validated genes from cotton mapping studies (expanded from Table 3)**

| **Gene** | **Trait** | **Mapping Approach** | **Validation Method** | **Key Finding** | **Reference** |
| --- | --- | --- | --- | --- | --- |
| *GhSAD1* | Cold tolerance | GWAS (200 accessions) | VIGS; Arabidopsis OE | Regulates ABA signaling; HapB increases cold tolerance | Ge et al., 2022 |
| *GhCHS* | Fiber elongation | eQTL mapping | RNAi in cotton | Flavonoid pathway; ROS accumulation affects elongation | Chen et al., 2026 |
| *GhDFR* | Secondary wall synthesis | eQTL mapping | RNAi in cotton | Flavonoid pathway; cellulose biosynthesis | Chen et al., 2026 |
| *GhPIN3* | Plant height | QTL mapping (BIL population) | VIGS | Auxin efflux carrier; silencing increases height | Ma et al., 2019 |
| *GhPAP* | Fiber strength | SLAF-BSA-seq + InDel analysis | VIGS; Arabidopsis OE | Plastid lipid-associated protein; affects helix formation | Zhang et al., 2025a |
| *GhABH* | Fiber quality (multi-effect) | Fine mapping + KASP | VIGS | α/β-hydrolase; regulates cell wall thickness | Zhang et al., 2025b |
| *GhTPS11* | Flowering time | QTL mapping + transcriptome | VIGS; Arabidopsis OE | Trehalose-6-phosphate synthase; age pathway | Feng et al., 2026 |
| *GhE6* | First fruiting branch height | RTM-GWAS + meta-QTL | VIGS | Early flowering regulation | Su et al., 2024 |
| *GhUBP15* | Plant height | UAV-based GWAS | VIGS | Ubiquitin protease; plant architecture | Ye et al., 2023 |
| *GhCUL1* | Plant height | UAV-based GWAS | VIGS | Cullin protein; plant architecture | Ye et al., 2023 |
| *GhCPR30* | Verticillium wilt resistance | QTL mapping + BSA-seq | VIGS | Disease resistance; silencing increases susceptibility | Wang et al., 2023 |
| *miR477b* | Fiber length | QTL co-localization | VIGS | Regulates DELLA via HOX3 | Song et al., 2024 |
| *GbCYP72A1* | Verticillium wilt resistance | QTL mapping | VIGS | Cytochrome P450; hormone signaling | Xu et al., 2023 |
| *GhALDH7B4* | Fiber strength | SLAF-BSA-seq | VIGS; Arabidopsis OE | Aldehyde dehydrogenase; cell wall components | Tang et al., 2024 |
| *GhRBB1_A07* | Fiber quality | MAGIC GWAS | Gene expression + SNP | Regeneration of bulb biogenesis; superior fiber | Islam et al., 2016 |
| *GhSusA1* | Fiber yield and quality | Candidate gene association | Transgenic overexpression | Sucrose synthase; sink strength regulation | Jiang et al., 2012 |
| *GhACO2* | Fiber elongation | Expression profiling | VIGS | ACC oxidase; ethylene synthesis | Various |
| *GhEXPA1* | Fiber elongation | QTL mapping + expression | VIGS | Expansin; cell wall loosening | Various |
| *GhHOX3* | Fiber length | Map-based cloning | Transgenic; mutants | Homeodomain transcription factor | Various |
| *GhMYB25-*like | Fiber initiation | Expression profiling | Transgenic; VIGS | MYB transcription factor; trichome development | Various |
| *GhACT_LI1* | Fiber elongation; plant architecture | Mapping-by-sequencing | Mutant analysis; VIGS | Actin; cytoskeleton organization | Thyssen et al., 2017; Cao et al., 2021 |
| *GhMML3_A12* | Fuzz fiber development | Association mapping | Mutant analysis | MYB-MIXTA-like transcription factor | Various |
| *GhTT2-3A* | Brown fiber pigmentation | Map-based cloning | Transgenic | Proanthocyanidin synthesis; fiber color | Yan et al., 2018 |
| *GhMYB25* | Fiber initiation | Expression profiling | Transgenic | MYB transcription factor; trichome initiation | Various |
| *GhMYB109* | Fiber initiation | Expression profiling | VIGS | MYB transcription factor; fiber development | Various |
| *GhHD1* | Stem trichome development | Map-based cloning | Mutant analysis | Homeodomain-leucine zipper; trichome initiation | Ding et al., 2015; Tang et al., 2021 |
| *GhTUB5* | Fiber elongation | Co-expression network + QTL | CRISPR; OE in Arabidopsis | Tubulin; cytoskeleton organization | Ma et al., 2024 |
| *GhPEL6* | Fiber length | QTL mapping (RIL population) | Candidate gene; expression | Pectin lyase; cell wall modification | Wang et al., 2020 |
| *GhCOBL4* | Lint percentage | QTL mapping (RIL population) | Candidate gene; expression | COBRA-like protein; cellulose deposition | Wang et al., 2020 |
| *GhMYB4* | Lint percentage | QTL mapping (RIL population) | Candidate gene; expression | MYB transcription factor | Wang et al., 2020 |
| *GhMYB85* | Lint percentage | QTL mapping (RIL population) | Candidate gene; expression | MYB transcription factor | Wang et al., 2020 |
| *GhCSLC6* | Fiber length | QTL mapping (RIL population) | Candidate gene; expression | Cellulose synthase-like protein | Wang et al., 2020 |
| *GhTBL5* | Fiber length | QTL mapping (RIL population) | Candidate gene; expression | Trichome birefringence-like protein | Wang et al., 2020 |
| *GhGASL3* | Fiber elongation | MAGIC GWAS | Candidate gene; expression | GAST-like protein; gibberellin response | Huang et al., 2021 |
| *GhACT1* | Fiber elongation | MAGIC GWAS | Candidate gene; expression | Actin; cytoskeleton | Huang et al., 2021 |
| *GhF6'H1* | Drought tolerance | BSA-seq + QTL mapping | VIGS | Flavonoid hydroxylase; stress response | Geng et al., 2024 |
| *Gh3AT1* | Drought tolerance | BSA-seq + QTL mapping | VIGS | Anthocyanin acyltransferase; stress response | Geng et al., 2024 |
| *GhPER55* | Drought tolerance | BSA-seq + QTL mapping | VIGS | Peroxidase; ROS homeostasis | Geng et al., 2024 |
| *Gh_A05G3226* | Lint percentage | QTL mapping (RIL population) | VIGS | Unknown; affects LP and FL | Guo et al., 2024 |
| *GhDAAT* | Salt tolerance | QTL mapping (RIL population) | VIGS | Diaminobutyrate acetyltransferase; salt response | Guo et al., 2024 |
| *GhHDG2* | Fiber micronaire | QTL fine mapping | Expression; TF binding | HD-ZIP IV transcription factor | Wang et al., 2025 |
| *GbGELP* | Fiber micronaire | QTL fine mapping | Heterologous in Arabidopsis | GDSL esterase/lipase; cell elongation | Wang et al., 2025 |
| *GbKAP* | Fiber uniformity | CSSL mapping | Heterologous in Arabidopsis | Keratin-associated protein; cell elongation | Wang et al., 2025 |

**Abbreviations:** VIGS, virus-induced gene silencing; OE, overexpression; BIL, backcross inbred line; BSA-seq, bulk segregant analysis sequencing; GWAS, genome-wide association study; UAV, unmanned aerial vehicle; RTM-GWAS, restricted two-stage multi-locus GWAS; CSSL, chromosome segment substitution line; LP, lint percentage; FL, fiber length; ROS, reactive oxygen species; TF, transcription factor.
