# Supplementary material for: Cotton genetic mapping for plant biotechnology: from markers to graph pan-genomes and sustainable breeding
Source: Front Plant Sci. 2026 May 12;17:1825852. doi: 10.3389/fpls.2026.1825852 (PMC13201509; doi:10.3389/fpls.2026.1825852)
Supplement: Supplementary Table 3 — Complete trait exemplars: where mapping most clearly touches sustainability (expanded from Table 4). [file Table3.docx]

**Supplementary Table S3. Complete trait exemplars: where mapping most clearly touches sustainability (expanded from Table 4)**

| **Trait Domain** | **Crop** | **Typical Targets** | **What Made It Hard** | **Mapping-to-Mechanism Bridge** | **Breeding Leverage (Sustainability Link)** | **Key References** |
| --- | --- | --- | --- | --- | --- | --- |
| Fiber quality | Cotton | QTL clusters; pleiotropy; stage-specific control; allele discovery via GWAS | Single-cell development; strong G×E; homoeolog redundancy | Fine-mapping + expression support; eQTL co-localization; SV-aware scans | Higher value per hectare; quality stability reduces waste and energy in processing | Mei et al., 2004; Abdurakhmonov et al., 2009; Sun et al., 2017; Liu et al., 2016; Xu et al., 2017; Zhang et al., 2025a; Zhang et al., 2025b; Tang et al., 2024; Islam et al., 2016 |
| Lint yield and stability | Cotton | Polygenic loci; environment-responsive QTL; yield-component dissection | Strong field heterogeneity; correlated traits; management effects | Multi-environment QTL + transcriptome-linked loci | Stable yield reduces land expansion pressure and input volatility | Yu et al., 2013; Liu et al., 2011 |
| Earliness/phenology and architecture | Cotton | Maturity SNPs; fruiting branch traits; flowering/earliness loci | Trade-offs with yield/fiber; photoperiod sensitivity; breeding-region stratification | GWAS + candidate genes; QTL-seq for key architectural traits | Earlier harvest windows reduce risk, irrigation needs, and pest pressure | Su et al., 2016; Huang et al., 2017; Zhang et al., 2021; Abdurakhmonov et al., 2007; Ma et al., 2019; Feng et al., 2026; Su et al., 2024 |
| Verticillium wilt resistance | Cotton | Major-effect loci plus quantitative background; resistance QTL clusters | Pathogen diversity; durability challenge; environment interactions | QTL clustering + fine mapping; integrative GWAS/QTL-seq/transcriptome → KASP markers | Durable resistance reduces fungicide use and yield losses | Jiang et al., 2009; Zhao et al., 2017; Zhang et al., 2013b; Abdelraheem et al., 2020; Zhao et al., 2021; Wang et al., 2023; Xu et al., 2023 |
| Fusarium wilt resistance | Cotton | Major resistance genes/loci; panel-wide QTL | Race structure and geographic spread | Gene mapping + GWAS confirmation in breeding germplasm | Reduced chemical inputs and replanting; resilience in infested soils | Wang et al., 2009; Abdelraheem et al., 2020 |
| Nematode resistance | Cotton | Resistance QTL for root-knot nematodes | Phenotyping destructive and environment-dependent | Interval/QTL mapping with validated markers | Reduced nematicide reliance; safer soils and water | Shen et al., 2006 |
| Abiotic resilience | Cotton | Meta-QTL for tolerance; expression-responsive loci | Strong G×E; drought timing effects; complex physiology | Meta-QTL synthesis; stress transcriptomes; eQTL and prediction | Water productivity and yield stability under climate volatility | Said et al., 2013; Zhang et al., 2013a; Abdelraheem et al., 2017; Ge et al., 2022 |
| Domestication and geographic differentiation | Cotton | Selection sweeps; cis-regulatory divergence; improvement loci | Bottlenecks; subgenome asymmetry; introgression vs local adaptation | Population genomics + regulatory divergence; mapping of improvement signals | Broadens adaptive diversity; guides pre-breeding for resilience | Wang et al., 2017a; He et al., 2021; Grover et al., 2020 |
| Pan-genomes, SV and graph coordinates | Cotton | SV-based GWAS; graph pan-genomes; structural haplotypes | Reference bias; SV genotyping; subgenome-aware coordinates | SV-aware association on pan-genomes; epigenome/3D-genome context | Captures hidden diversity and reduces false negatives; enables durable stacking | Jin et al., 2023; Zhang et al., 2026; Yang et al., 2026; Huang et al., 2024 |
| Multi-parent resources (MAGIC) | Cotton | Allele-series mapping; recombination-rich panels | Construction cost; phenotyping scale | MAGIC GWAS; bridging linkage and association | Accelerates discovery and validation in breeding-relevant backgrounds | Huang et al., 2021 |
| Grain yield and stability | Wheat | Yield QTL; thousand-kernel weight; yield components | Large genome; polyploidy; G×E complexity | Multi-environment GWAS; meta-QTL; integration with GS | Stable yield reduces land expansion pressure | Chidzanga et al., 2022; Tyrka et al., 2023; López-Fernández et al., 2023 |
| End-use quality | Wheat | Dough rheology; baking quality; protein content | Complex traits; multiple components; G×E interaction | GWAS + genomic prediction; candidate gene validation | Higher value; reduced processing waste | Gill et al., 2025 |
| Pre-harvest sprouting resistance | Wheat | Seed dormancy; germination traits | Complex genetics; environmental sensitivity | Pan-genome-enabled GWAS; structural variant detection | Reduced losses; improved food security | Dallinger et al., 2024 |
| Grain shape | Rice | Grain length; grain width; grain weight | Multiple genes; pleiotropy | Fine-mapping; candidate gene validation | Market preference; higher value | Feng et al., 2016 |
| Salinity tolerance | Rice | Seedling survival; ion homeostasis; recovery traits | Complex physiology; G×E interaction | Multi-environment GWAS; haplotype analysis | Expanded cultivation area; yield stability | Siddique et al., 2025 |
| Drought tolerance | Rice | Root architecture; canopy temperature; yield under stress | Complex traits; measurement challenges | GWAS in landraces; candidate gene identification | Water productivity; climate resilience | Beena et al., 2021 |
| Aflatoxin resistance | Maize | Mycotoxin accumulation; fungal resistance | Complex inheritance; phenotyping difficulty | QTL mapping + GWAS; meta-analysis | Reduced contamination; food safety | Zhang et al., 2016 |
| Flowering time | Maize | Days to anthesis; photoperiod response | Complex genetics; population structure | NAM population GWAS; candidate gene validation | Adaptation to diverse environments | Buckler et al., 2009 |
| Powdery mildew resistance | Barley | Seedling resistance; adult plant resistance | Quantitative resistance; multiple loci | Multi-environment GWAS; small-effect QTL detection | Reduced fungicide use | Guo et al., 2024 |
| Drought tolerance | Barley | Seedling vigor; recovery under osmotic stress | Complex physiology; phenotyping bottlenecks | Multi-environment GWAS; candidate gene identification | Water productivity; climate resilience | Slawin et al., 2024 |
| Isoflavone content | Soybean | Seed isoflavone concentration | Complex biosynthesis; G×E interaction | GWAS + linkage mapping; functional validation | Nutritional enhancement; health benefits | Wu et al., 2020 |
| Salt stress response | Soybean | Germination; seedling growth under salinity | Complex response; multiple pathways | Association mapping; functional validation via VIGS | Expanded cultivation; yield stability | Liao et al., 2021 |
| Iron and zinc concentration | Lentil | Seed mineral content | Complex genetics; phenotyping cost | Multi-environment GWAS; marker validation | Nutritional enhancement; human health | Singh et al., 2017 |
| Tocopherol synthesis | Arabidopsis | Vitamin E content | Complex pathway; multiple genes | GWAS + mutant analysis; transgenic validation | Mechanistic understanding; translational potential | Albert et al., 2022 |
| Seed dormancy | Arabidopsis | Germination timing | Complex regulation; environmental interaction | eQTL + mutant analysis; regulatory network | Understanding trait architecture | Yano et al., 2013 |
| Plant-microbiome interactions | Arabidopsis | Microbiome composition; community structure | Complex interactions; high-dimensional data | Microbiome GWAS; systems biology | Soil health; disease suppression | Beilsmith et al., 2019 |
| Biomass and sugar yield | Sorghum | Biomass accumulation; sugar content | Complex traits; perennial habit | GWAS + transgenic validation; ortholog identification | Bioenergy improvement | Upadhyaya et al., 2022 |
| Inflorescence morphology | Sorghum | Panicle architecture; seed set | Complex development; multiple genes | NAM population GWAS; candidate gene mapping | Yield improvement | Olatoye et al., 2020 |
| Cold tolerance | Sorghum | Germination under cold; seedling vigor | Complex physiology; phenotyping challenges | Multi-environment GWAS; QTL validation | Expanded growing season | Upadhyaya et al., 2016 |
| Root and shoot architecture | Barley | Seedling root traits; early vigor | Phenotyping difficulty; complex genetics | GWAS in diversity panel; QTL hotspot identification | Improved establishment; stress tolerance | Abdel-Ghani et al., 2019 |
